# Exploring Schwann Cell Behavior on Electrospun Polyhydroxybutyrate Scaffolds with Varied Pore Sizes and Fiber Thicknesses: Implications for Neural Tissue Engineering

**DOI:** 10.3390/polym15244625

**Published:** 2023-12-06

**Authors:** María Florencia Lezcano, Paulina Martínez-Rodríguez, Karina Godoy, Jeyson Hermosilla, Francisca Acevedo, Iván Emilio Gareis, Fernando José Dias

**Affiliations:** 1 Departamento de Bioingeniería, Facultad de Ingeniería, Universidad Nacional de Entre Ríos, Oro Verde 3100, Argentinaigareis@ingenieria.uner.edu.ar (I.E.G.); 2 Oral Biology Research Centre (CIBO-UFRO), Department of Integral Adults Dentistry, Dental School, Universidad de La Frontera, Temuco 4780000, Chile; 3 Scientific and Technological Bioresource Nucleus (BIOREN-UFRO), Universidad de La Frontera, Temuco 4780000, Chile; 4 Programa de doctorado en Ciencias de Recursos Naturales, Universidad de La Frontera, Temuco 4780000, Chile; 5 Center of Excellence in Translational Medicine (CEMT), Faculty of Medicine, Scientific and Technological Bioresource Nucleus (BIOREN), Universidad de La Frontera, Temuco 4780000, Chile; francisca.acevedo@ufrontera.cl; 6 Department of Basic Sciences, Faculty of Medicine, Universidad de La Frontera, Temuco 4780000, Chile

**Keywords:** Schwann cell, polyhydroxybutyrate, electrospinning, fiber thickness, pore size

## Abstract

The placement of a polymeric electrospun scaffold is among the most promising strategies to improve nerve regeneration after critical neurotmesis. It is of great interest to investigate the effect of these structures on Schwann cells (SCs), as these cells lead nerve regeneration and functional recovery. The aim of this study was to assess SC viability and morphology when cultured on polyhydroxybutyrate (PHB) electrospun scaffolds with varied microfiber thicknesses and pore sizes. Six electrospun scaffolds were obtained using different PHB solutions and electrospinning parameters. All the scaffolds were morphologically characterized in terms of fiber thickness, pore size, and overall appearance by analyzing their SEM images. SCs seeded onto the scaffolds were analyzed in terms of viability and morphology throughout the culture period through MTT assay and SEM imaging. The SCs were cultured on three scaffolds with homogeneous smooth fibers (fiber thicknesses: 2.4 μm, 3.1 μm, and 4.3 μm; pore sizes: 16.7 μm, 22.4 μm, and 27.8 μm). SC infiltration and adhesion resulted in the formation of a three-dimensional network composed of intertwined fibers and cells. The SCs attached to the scaffolds maintained their characteristic shape and size throughout the culture period. Bigger pores and thicker fibers resulted in higher SC viability.

## 1. Introduction

Nerves are fragile and can be damaged as a result of pressure, stretching, cutting, and poor blood supply [1]. Peripheral nerve injury is a problem of high incidence that causes loss of motor function and sensibility, often resulting in life-long disability [1,2]. When the injury results in a small nerve gap, it can be closed by suturing the ends of the nerve. However, when the gap is too large, the most common approach is to close it, placing an autograft or an allograft, causing denervation or requiring a donor [2,3]. An interesting alternative to close extensive gaps is the placement of a biocompatible and biodegradable artificial scaffold with the capability of promoting nerve regeneration [2].

One of the major challenges in developing a scaffold is choosing the adequate materials [4]. In the last two decades, artificial scaffolds for nerve regeneration have been produced using a variety of polymers, both natural and synthetic and biodegradable or non-biodegradable [5]. Among the bioresorbable materials, aliphatic polyesters and copolyesters have been frequently used for nerve regeneration. Examples include poly(L-lactic acid) (PLLA), poly(glycolic acid) (PGA), poly(lactic acid-e-caprolactone), poly(DL-lactide-co-glycolide), poly(1,3-trimethylenecarbonate-e-caprolactone), and poly(caprolactone) (PCL) [3]. Among the biodegradable and biocompatible polymers, poly-hydroxybutyrate (PHB) stands out as it is an FDA-approved polymer synthesized from renewable sources, having a positive social and environmental impact [6,7,8]. It is also a versatile natural polymer that can be extruded, molded, made into films, and spun into fibers [9].

Among the currently available fabrication techniques, electrospinning is one of the most promising methods to obtain polymeric scaffolds for tissue engineering [10]. Electrospinning is a fiber production method that uses electric force to draw charged threads of polymer solutions, obtaining ultrathin fibers. Thanks to their nano- and microfibers, large surface areas, and superior mechanical properties, electrospun scaffolds can mimic the hierarchical structure present in the extracellular matrix providing structural support for cell attachment and subsequent tissue development [10,11]. One of the most important parameters of this kind of design is the diameter of the fibers, which has a considerable impact not only on the degradation rate of the material but also on cell behavior [12,13,14,15]. Other important features are the porosity and the pore size, which modulate permeability, exchange of nutrients, retention of neurotrophic factors, mechanical properties, degradability, and vascularization [16]. The regulation of the scaffold pore size is a critical factor in regenerative therapy [17]. In order to achieve satisfactory results in tissue engineering, the pore size should be adjusted to a size dependent on the specific cells being cultured [18,19]. The successful application of scaffolds in regenerative medicine is dependent on complex pore-size- and cell-type-dependent processes and needs individual experimental optimization considering the regenerative and therapeutic goals [17]. The diameter of electrospun fibers may vary according to polymer concentration, viscosity, and molecular weight [20,21]. Different studies have reported that the fiber diameter can alter cell morphology, proliferation, and migration [22,23]. It has also been reported that neuronal cells exhibited greater growth, alignment, and differentiation with fibers ranging from 1.3 µm to 30 µm [13,14,21,22,24] than with fibers smaller than 0.2 µm [14]. These results reveal that the diameter of the fiber influences nerve regeneration. However, there is still no consensus regarding the most appropriate fiber diameter for the construction of a fibrous scaffold for nerve regeneration using the electrospinning technique [25].

In terms of neural tissue engineering, it is of great interest to investigate the effect that these scaffolds have on Schwann cells (SCs) as these cells lead nerve regeneration and functional recovery [26]. Following nerve damage, SCs from both the proximal and the distal nerve stumps migrate into the nerve bridge and form SC cords to guide axon regeneration [27]. SCs modulate and drive the regeneration process by promoting neuronal survival, damaged axon disintegration, myelin clearance, axonal regrowth and guidance to their former target, and finally by remyelinating the regenerated axon [26]. During this process, they not only migrate into the area of damaged tissue and become a key component of the regenerating tissue but also secrete signaling molecules to attract macrophages, support neuronal survival, promote axonal regrowth, activate local mesenchymal stem cells, and interact with other cell types [27]. One of the key features of the specialized repair SCs is that they become highly motile, which allows them to reach damaged tissue and lead regeneration [27]. This characteristic motility of repair SCs can be modulated by scaffold material and fiber thickness [12,13,14,15]. The current research shows that peripheral-nerve-associated SCs possess the capacity to promote the repair of multiple other tissues including skin wound healing, digit tip repair, and tooth regeneration [27]. Therefore, studying the behavior of these cells in close relationship with the environment could help to improve not only nerve regeneration but also the repair of other kinds of tissues.

The aim of this study was to assess SC viability and morphology when cultured on PHB electrospun scaffolds with varied microfiber thicknesses and pore sizes.

## 2. Materials and Methods

### 2.1. Scaffold Fabrication

Commercial PHB (poly[(R)-3-hydroxybutyric acid], Sigma Aldrich, St. Louis, MO, USA) was dissolved in chloroform at 60 °C for 24 h. The PHB used had a microbial origin and a molecular weight of 300 kDa [28]. Chloroform is an organic solvent widely used to solubilize polymers for electrospinning. In a previous work [29], it was shown that it allows the formation of PHB electrospun fibers free of artifacts such as beads and agglomerations. 

Six different electrospun scaffolds were obtained using different PHB solutions and different parameters for the electrospinning machine (NEU-BM, Shenzhen Tong Li Tech Co., Ltd., Shenzhen, China). The electrospinning parameters were selected based on a previous work [28] with some modifications derived from unpublished tests. These parameters are presented in Table 1 (only the combinations that led to fully formed scaffolds are shown).

### 2.2. Scaffold Morphological Characterization

**Figure 1:**
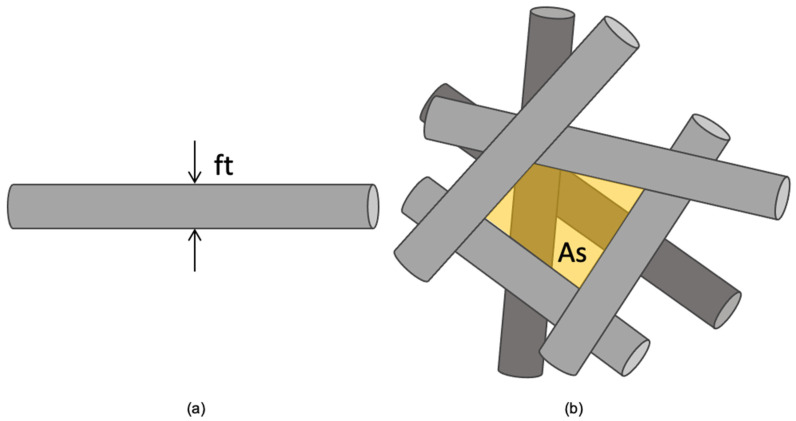
Schematic of SEM image analysis through ImageJ: (**a**) fiber thickness (ft); (**b**) free space between superficial fibers (As, shaded area).

All the scaffolds were morphologically characterized by analyzing SEM images taken with a variable-pressure scanning electron microscope (STEM SU-3500, Hitachi, Tokyo, Japan). The images were processed using open-source software (ImageJ 1.53, National Institute of Health, Bethesda, MD, USA), through which, for each scaffold, the thicknesses of 30 superficial fibers (ft) and the areas of 30 free spaces between superficial fibers (As) were measured as schematized in Figure 1. The pore size was then calculated as the effective pore diameter by applying Equation (1).

(1) $$PoreSize=2\sqrt{\frac{As}{\pi }}$$

### 2.3. Cell-Scaffold Imaging

Scaffolds incubated with cells were fixed with 2.5% glutaraldehyde in 0.1 M Sørensen’s phosphate buffer (Electron Microscopy Sciences, Hatfield, PA, USA) for 30 min and analyzed in an environmental scanning electron microscope (SU3500, Hitachi, Japan) in low-vacuum conditions. The morphology of the attached cells was analyzed.

### 2.4. Cell Viability Assay

Only scaffolds with homogeneous fibers were selected for this analysis. A total of 5 × 10^3^ SCs (SCL 4.1/F7, ECACC 93031204) were seeded onto circular-shaped scaffolds (5 mm diameter) in technical triplicates in a 96-well plate. After 2 h of incubation, 100 μL of Ham’s F12 supplemented with 10% of FBS and 1% P/S was added. The scaffolds were incubated for 1, 3, 5, and 7 days in a humid environment at 37 °C, 5% CO_2_. The cell viability was assessed through an MTT assay (Proliferation Kit I, Roche, Indianapolis, IN, US). Optical density readings were performed on a microplate reader at λ = 570 nm (Infinite^®^ F50; Tecan, Männedorf, Switzerland). The tests were performed in triplicates.

### 2.5. Statistical Analysis

Descriptive statistics (mean and standard deviation) were conducted to analyze the morphometric characteristics of the scaffolds. The Matplotlib and Seaborn open-source modules for Python were used to obtain proliferation curves, kernel density estimate plots, and box plots. The Scipy and Statsmodels open-source modules for Python were used to test the normality of the data and conduct one-way (to compare pore size and fiber thickness among scaffolds) and two-way (to compare cell viability among scaffolds and across time) ANOVA tests, with t-test and Bonferroni correction as post hoc tests. The morphometric data (pore size and fiber thickness) were correlated using Pearson’s method. A statistical significance of α = 0.05 was used.

## 3. Results

### 3.1. PHB Scaffold Morphology

**Figure 2:**
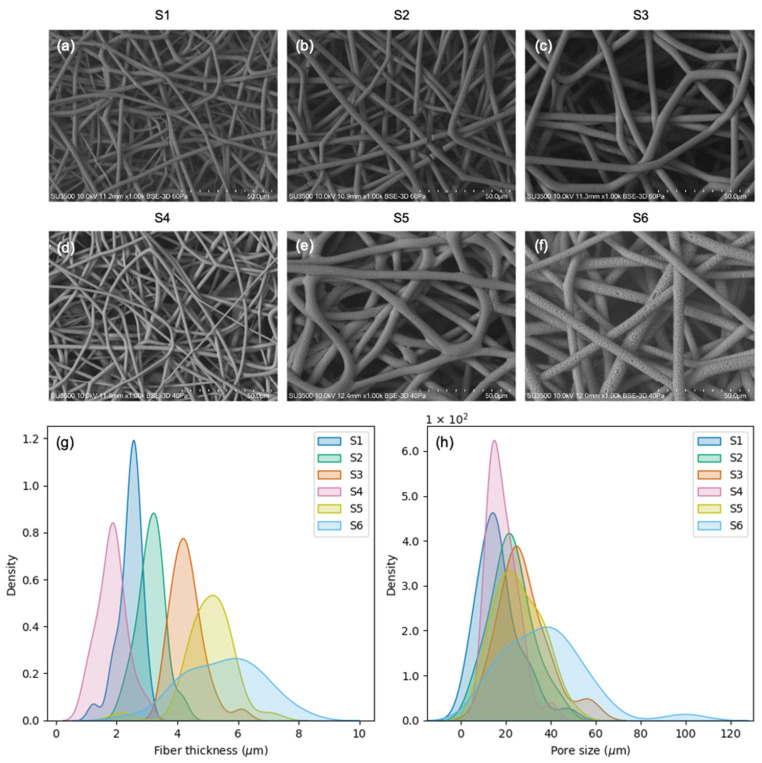
Scaffold morphologic characterization: (**a**–**f**) SEM images of all the electrospun scaffolds obtained; (**g**) kernel density estimate plots for fiber thickness; (**h**) kernel density estimate plots for pore size.

The SEM images of the scaffolds (Figure 2a–f) revealed that some samples (S1, S2, and S3) presented smooth fibers, while others (S5 and S6) presented rough fibers, and one of them (S4) presented a combination of both. In terms of fiber thickness (Figure 2g; Table 2), all the scaffolds presented significant differences (*p* ≤ 0.05) with the exception of S5 and S6, which had equivalent fibers. In terms of pore size (Figure 2h; Table 2), few statistical differences were found. Different distributions for the pore size were found in the following pairs of scaffolds: S1–S3 (*p* = 0.02), S1–S6 (*p* = 0.00), S2–S6 (*p* = 0.03), S3–S4 (*p* = 0.01), S4–S5 (*p* = 0.04), and S4–S6 (*p* = 0.00). S6 displayed the highest values and the highest variability for both fiber thickness and pore size (Table 2). All the measurements are available in Tables S1 (pore size) and S2 (fiber thickness).

**Figure 3:**
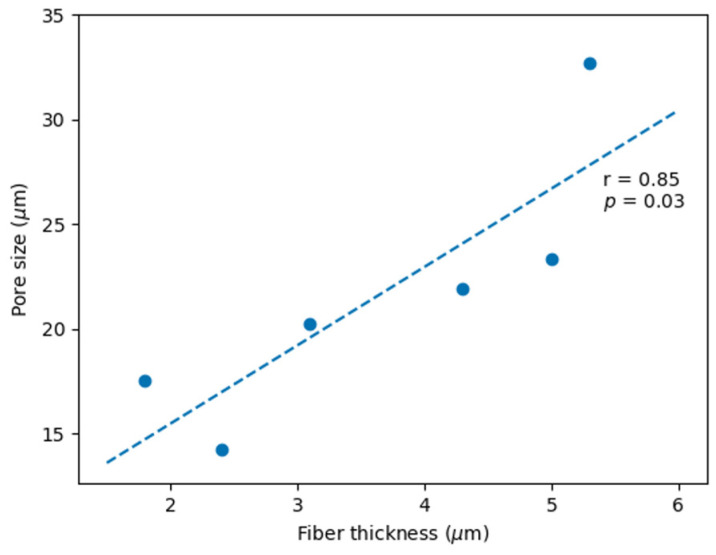
Pearson correlation between pore size and fiber thickness (r = 0.85, *p* = 0.03).

Increasing the flow rate resulted in thicker fibers, bigger pores, and higher variability. Increasing the voltage resulted in either thicker or thinner fibers, depending on the PHB concentration. The same was observed for pore size. When increasing the PHB concentration, the scaffolds began to present rough and thicker fibers, sometimes mixed with thinner smooth fibers. A very high correlation (r = 0.85, *p* = 0.03; Figure 3) between the pore size and the fiber thickness was obtained by manipulating the PHB concentration, electrospinning voltage, and flow rate.

### 3.2. Viability and Morphology of SCs Attached to Scaffolds with Varied Pore Sizes and Fiber Thicknesses

**Figure 4:**
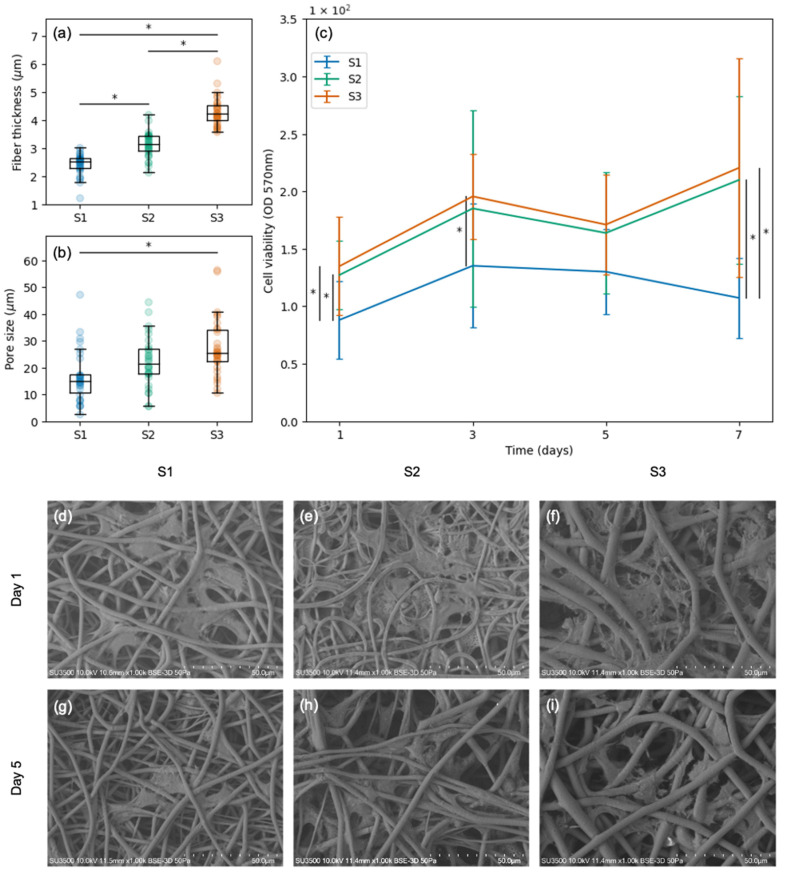
SCs cultured on three different scaffolds (S1, S2, and S3): (**a**) fiber thickness distribution of each scaffold; (**b**) pore size distribution of each scaffold; (**c**) cell viability assessment at 570 nm absorbance on days 1, 3, 5, and 7; (**d**–**f**) SCs attached to S1, S2, and S3 on day 1; (**g**–**i**) SCs attached to S1, S2, and S3 on day 5. Significant statistical differences (*p* ≤ 0.05) are marked with *.

Scaffolds S1, S2, and S3 were selected to conduct SC viability tests. These scaffolds presented smooth fibers (Figure 2a–c), different fiber thicknesses (Figure 4a), and varied pore sizes (Figure 4b). Regarding fiber thickness, S1 had thinner fibers than S2 (*p* = 0.00) and S3 (*p* = 0.00); likewise, S2 had thinner fibers than S3 (*p* = 0.00). Regarding pore size, S1 had smaller pores than S3 (*p* = 0.00); however, S2 and S3 did not present significant differences. In terms of cell viability, significant differences were found between S1 (the scaffold with the smallest fibers and the smallest pores) and the rest of the scaffolds throughout the incubation period (days 1, 3, and 7; Figure 4c). Bigger pores and thicker fibers resulted in higher cell viability. SEM images revealed that the SCs seeded on scaffolds S1, S2, and S3 penetrated the porous structure and adhered to the material. This resulted in the formation of a three-dimensional network composed of intertwined fibers and cells. The number of cells did not change significantly throughout the culture period, and the cells maintained their characteristic shape and size (days 1 and 5 shown in Figure 4d–i). All the cell viability measurements are available in Table S3.

## 4. Discussion

In the present study, the viability and morphology of SCs cultured on PHB electrospun scaffolds with varied fiber thicknesses and pore sizes were assessed. Six different microfibrous electrospun scaffolds were obtained by varying the PHB concentration and the electrospinning parameters. Three of these scaffolds were ruled out due to the presence of heterogeneous rough fibers. The rest of the scaffolds (fiber thicknesses: 2.4 μm, 3.1 μm, and 4.3 μm; pore sizes: 16.7 μm, 22.4 μm, and 27.8 μm) were seeded with SCs. In all cases, the cells penetrated the porous structure and adhered to the material, maintaining their characteristic shape and size throughout seven days of incubation. Differences in the cell viability were analyzed in relation to fiber thickness and pore size. Bigger pores and thicker fibers resulted in higher cell viability.

The obtained results show that SC infiltration and adhesion resulted in the formation of a three-dimensional network composed of intertwined fibers and cells, creating an environment that mimics the natural extracellular matrix found in tissues. This in vitro feature is highly advantageous in itself since cell-seeded scaffolds can increase their effectiveness [30]. In the case of nerve regeneration, it is of great interest to seed SCs onto scaffolds, as these cells not only elaborate neurotrophic factors and other proteins that have a positive effect on nerve growth but they also express surface proteins that may propel the newly extending axons forward along a carpet of SCs [31]. PHB scaffolds seeded with SCs have already been tested in rodents, obtaining promising results for peripheral nerve regeneration [32,33,34,35] and spinal cord repair [36,37]. However, further studies are needed to identify and understand the underlying mechanisms involved in the process. In the present study, in vitro experiments enabled an analysis of the effect of PHB fiber thickness and pore size on SC viability and morphology as the advantage of in vitro experiments is that they supplement in vivo studies by permitting a controlled and detailed examination of specific cellular mechanisms [38]. Two of the scaffolds seeded with SCs had equivalent pores and one of them had significantly smaller pores. The scaffolds with equivalent pore size presented equivalent cell viability. The scaffold with smaller pore size presented significantly lower cell viability than the scaffolds with bigger pores.

SEM analysis in the present study revealed that SCs adhered to microfibrous scaffolds align themselves along and around the microfibers. In this case, the three scaffolds seeded with SCs had different fiber thicknesses. The thickest fibers presented the highest cell viability. The two scaffolds with the thinnest fibers presented the lowest cell viability. However, it is not clear w this behavior is a response to pore size or fiber thickness since the variables controlled in the present study did not allow the pore size to change while maintaining fiber thickness. Furthermore, the pore sizes and fiber thicknesses analyzed in the present study presented a very high correlation. The same behavior has been reported previously for different polymers including PCL, gelatin, and tropoelastin [39]. The effect of PHB fiber diameter on SCs has not been found in the literature. However, one study tested the effect of gelatin nano- and microfibers on SC adhesion and proliferation, finding that migration rate and motility were greater in cells cultured on microfibers [12]. Their results suggest that the topography of electrospun gelatin fibers can be adjusted to modulate SC and axon organization and that both nano- and microfibers are promising fillers for scaffolds for peripheral nerve repair [12]. In the present study, the fibers within the obtained scaffolds may not only provide physical support but also serve as guidance cues for cell movement and alignment. 

In the present study, the biocompatibility of electrospun PHB scaffolds made of microfibers has been assessed by seeding cells onto the scaffolds. PHB can cause prolonged and acute inflammatory responses, so it is important to obtain a high-purity material to build carefully designed scaffolds and always check their biocompatibility [40]. The first film for surgical applications made of PHB was approved by the FDA in 2007 [41,42]. The same year, Suwantong et al. (2007) [43] published an in vitro study in which they assessed the cytotoxicity of PHB fiber mats seeded with SCs, showing that the material is compatible with this kind of cell [43]. However, no modifications were made to the mats in terms of fibers and pores in order to test their influence in SC behavior as made in the present study. PHB represents a great industrial and scientific advance in the search for new sustainable energy sources; however, one of the main challenges faced by researchers is the design of scaffolds with desirable properties for the growth and proliferation of cells [44]. The scaffold designs tested in the present study have demonstrated a positive effect on SC growth and proliferation, especially the ones with the thickest fibers and biggest pores.

In the present study, the MTT assay results indirectly suggest that the number of active SCs attached to the scaffolds did not change significantly throughout the culture period. This apparent reduction in the proliferation rate could be explained by the pronounced contact inhibition of the growth of the used cells (SCL 4.1/F7). The pore size was large enough for the cells to infiltrate the scaffold, being surrounded by fibers with no space to divide. Regarding the cell morphology, it would be nice to observe the morphology of seeded cells over a longer time period since, in the present study, only 5 days were analyzed. Regarding translational aspects, rodent SCs, such as the ones used in this study, are notoriously more indiscriminate than human SCs; it has been reported that they can accept different substrates and divide in response to a wider variety of growth factors [45]. Therefore, even though the findings reported in the present study are promising, they should not be generalized to all species. Even though the present study found potential evidence that bigger pores and thicker PHB fibers result in higher SC viability, further studies are needed to optimize the design of PHB electrospun scaffolds for nerve regeneration.

## 5. Conclusions

Electrospun PHB microfibrous scaffolds proved to be compatible with SCs. SC infiltration and adhesion resulted in the formation of a three-dimensional network composed of intertwined fibers and cells, creating an environment that mimics the natural extracellular matrix found in tissues. The cell viability did not change for different fiber thicknesses when pore sizes were equivalent. The cell viability only changed when the pore size changed. Although we were unable to obtain scaffolds with different pore sizes and equivalent pore sizes, our results suggest that the pore size has a dominant influence on the cell viability. The number of active SCs attached to the scaffolds did not change significantly throughout a culture period of seven days.

## Figures and Tables

**Table 1:** Parameters for scaffold fabrication.

Scaffold	PHB (%)	Voltage (kV)	Flow Rate (mL/h)	Needle–Collector Distance (cm)	Collector Diameter (cm)	Collector Speed (rpm)	Time (min)
S1	10	25	1	15	3.1	50	150
S2	10	20	1	15	3.1	50	150
S3	15	25	1	15	3.1	50	150
S4	15	20	1	15	3.1	50	150
S5	15	25	2	15	3.1	50	150
S6	15	20	2	15	3.1	50	150

**Table 2:** Fiber thickness and pore size.

Scaffold	Fiber Thickness (μm)	Pore Size (μm)
S1	2.4 ± 0.4	16.7 ± 9.7
S2	3.1 ± 0.4	22.4 ± 9.4
S3	4.3 ± 0.5	27.8 ± 11.0
S4	1.8 ± 0.5	18.5 ± 6.6
S5	5.0 ± 0.8	25.5 ± 10.3
S6	5.3 ± 1.3	37.0 ± 18.5

## Data Availability

The data presented in this study are available in the Supplementary Materials.

## Supplementary Materials

The following supporting information can be downloaded at https://www.mdpi.com/article/10.3390/polym15244625/s1: Table S1: Pore size; Table S2: Fiber thickness; Table S3: Cell viability.
